# Evaluation of expression of vitamin D receptor related lncRNAs in lung cancer

**DOI:** 10.1016/j.ncrna.2020.05.001

**Published:** 2020-05-24

**Authors:** Tahereh Gheliji, Vahid Kholghi Oskooei, Asghar Ashrafi Hafez, Mohammad Taheri, Soudeh Ghafouri-Fard

**Affiliations:** a Department of Biology, International Pardis, University of Guilan, Rasht, Iran; b Department of Laboratory Sciences, School of Paramedical Sciences, Torbat Heydariyeh University of Medical Sciences, Torbat Heydariyeh, Iran; c Neuroscience Research Center, Torbat Heydariyeh University of Medical Sciences, Torbat Heydariyeh, Iran; d Cancer Research Center, Shahid Beheshti University of Medical Sciences, Tehran, Iran; e Urogenital Stem Cell Research Center, Shahid Beheshti University of Medical Sciences, Tehran, Iran; f Department of Medical Genetics, Shahid Beheshti University of Medical Sciences, Tehran, Iran

**Keywords:** Lung cancer, lncRNA, Vitamin D receptor

## Abstract

Lung cancer as the most common cancer in the world is associated with high rate of mortality. Previous studies have detected expression of vitamin D receptor (VDR) in lung cancer tissues and reported significant of this gene in determination of patients' survival. Methods: In the current study, we assessed expression of *VDR* and five long non-coding RNAs (lncRNAs) which have been associated with VDR (*MALAT1*, *SNHG16*, *SNHG6*, *LINC00346, LINC00511*) in 32 pairs of lung cancer tissues and adjacent non-cancerous tissues (ANCTs) using real time PCR method. Expression of *VDR* was significantly decreased in tumor tissues obtained from male patients compared with their matched ANCTs (ER = 0.31, P value = 0.02). However, this pattern was not detected in female subjects (ER = 0.93, P value = 0.94). Expression of LINC00346 was significantly decreased in tumoral tissues compared with ANCTs (Expression ratio (ER) = 0.38, P value = 0.03). When evaluating expression of this lncRNA based on the sex of patients, differences in its expression was only significant among males (ER = 0.3, P value = 0.04). *VDR* expression was significantly associated with sex of patients in a way that most male patients exhibited down-regulation of this gene in their tumor tissue samples compared with the paired ANCTs (P = 0.03). Expression levels of LINC00346 could discriminate lung cancer tissues from ANCTs with sensitivity of 83.3% and specificity of 52.4%. Correlations between expressions of *SNHG6* and other genes were all significant in tumoral tissues but insignificant in ANCTs. The current investigation potentiates *VDR* and LINC00346 as possible participants in the pathogenesis of lung cancer.

## 1 Introduction

Lung cancer is the foremost common cancer in the world and the chief source of cancer-related death [1]. According to the GLOBOCAN report, this malignancy comprise almost one fifth of cancer mortalities [1]. Lung cancer is histologically categorized into two principal subgroups: small cell lung carcinoma and non-small cell carcinoma (NSCLC) with the latter encompassing 85% of all lung cancer cases. NSCLC is subdivided into adenocarcinoma (AC), squamous cell carcinoma (SCC) and large cell carcinoma [2]. Previous studies have reported expression of vitamin D receptor (VDR) in both SCC and AC tissues and speculated the possibility of response of cancerous tissues to the differentiating effects of vitamin D analogues [3]. More recent studies have shown correlation between high VDR expression and better patients' outcome in lung cancer. They also reported association between anti-proliferative effect of vitamin D and level of expression of VDR in cell lines [4]. The effects of *VDR* in the pathogenesis of lung cancer has been further highlighted by the observed associations between several single nucleotide polymorphisms within this gene and risk of lung cancer [5]. The anti-cancer effects of VDR has been shown to be exerted through different mechanisms among them are modulating expression of cancer-associated long non-coding RNAs (lncRNAs) [6]. Moreover, vitamin D response elements (VDREs) have been identified in genomic regions that encode lncRNAs [7]. Through a novel bioinformatics approach, Kholghi Oskooei et al. have previously identified *MALAT1*, *SNHG16*, *SNHG6*, *LINC00346* and *LINC00511* as VDR-associated lncRNAs in breast cancer [8]. Assessment of expression of *VDR* and these lncRNAs in breast cancer tissues and adjacent non-cancerous tissues (ANCTs) has led to identification of aberrant expression of *MALAT1* and *LINC00511* in tumoral specimens [8]. In the current study, we appraised expression of *VDR* and these lncRNAs in lung cancer tissues and ANCTs to evaluate the role of VDR-associated lncRNAs in this type of human malignancy.

## 2 Materials and methods

### 2.1 Patients

The current study was performed on tissues samples obtained from 32 patients with lung cancer. Both tumoral tissues and ANCTs were obtained from each patient during surgical removal of tumors [9]. Samples were obtained from Labbafinejad Hospital during 2017–2018. All specimens were assessed by a pathologist to appraise the presence of cancer cells. No chemo/radiotherapy was performed before surgery for any of patients. Informed consent forms were obtained from all study participants. The study protocol was approved by the ethical committee of Shahid Beheshti University of Medical Sciences (IR.SBMU.MSP.REC.1395.525).

### 2.2 Expression assays

Expressions of *VDR* and five associated lncRNAs (*MALAT1*, *SNHG16*, *SNHG6*, *LINC00346, LINC00511*) were compared between lung cancer tissues (n = 32) and ANCTs (n = 32) using real time PCR method. At first, total RNA was isolated from all specimens using TRIzol™ Reagent (Invitrogen, Carlsbad, CA, USA). To avoid amplification of DNA remnants in further steps, extracted RNAs were treated by DNase I (Thermo Scientific, Lithuania). Subsequently, cDNA was produced from RNA samples by using the OneStep RT-PCR Series Kit (BioFact™, Seoul, South Korea). The relative expression of *VDR* and mentioned lncRNAs was measured using RealQ Plus 2x PCR Master Mix (Ampliqon, Odense, Denmark). Experiments were performed in the rotor gene 6000 cycler. *B2M* gene was used as the reference gene. Primer sequences and PCR conditions were similar to the previous study by Kholghi et al. [8].

### 2.3 Statistical analyses

Statistical analyses were performed in SPSS v.20 (IBM Corp., Armonk, NY, USA). The difference in expression of mentioned genes between lung cancer tissues and ANCTs was judged using paired t-test. Analyses were performed considering PCR efficiencies of all reactions. Chi-square test was used to evaluate association between patients' information and relative expression of genes. Correlations between relative expressions of genes were appraised using regression model. Level of significance was set P values < 0.05. Diagnostic power of mentioned genes was assessed through depicting receiver operating characteristic (ROC) curve and calculation of the area under curve (AUC) levels.

## 3 Results

### 3.1 General characteristics of lung cancer patients

Table 1 shows the general data of lung cancer patients.

**Table 1:** General data of patients (AC: adenocarcinoma, SCC: squamous cell carcinoma).

Parameters		Values
Age (Mean ± standard deviation (range))		57 ± 8.78 (37-80)
Gender (%)	Male	71.4%
	Female	28.6%
Subtype (%)	AC	57.1%
	SCC	42.9%
Stage (%)	I	19%
	II	38.1%
	III	42.9%
Smoking (%)	Yes	19%
	No	81%

### 3.2 Expression assays

**Fig. 1:**
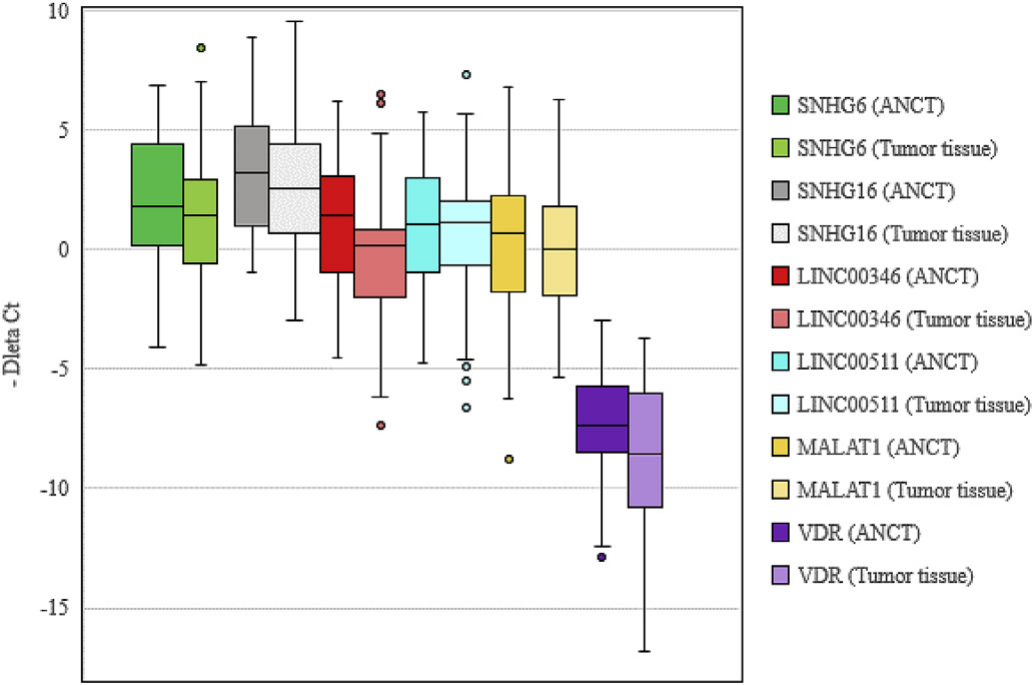
Expression of *VDR* and its related lncRNAs in lung cancer tissues and ANCTs.

Expression of *LINC00346* was significantly decreased in tumoral tissues compared with ANCTs (Expression ratio (ER) = 0.38, P value = 0.03). When evaluating expression of this lncRNA based on the sex of patients, differences in its expression was only significant among males (ER = 0.3, P value = 0.04). In addition, expression of *VDR* was significantly decreased in tumor tissues obtained from male patients compared with their matched ANCTs (ER = 0.31, P value = 0.02). However, this pattern was not detected in female subjects (ER = 0.93, P value = 0.94). Fig. 1 and Table 2 show the details of expression analyses.

**Table 2:** Relative expression of VDR and lncRNAs in lung cancer tissues compared with ANCTs.

Genes	Parameters	Total patients (n = 42)	Male patient (n = 31)	Female patients (n = 11)
*SNHG6*	Expression ratio	0.61	0.4	1.98
	P-value	0.25	0.08	0.38
*SNHG16*	Expression ratio	0.49	0.42	0.78
	P-value	0.11	0.09	0.79
*LINC00346*	Expression ratio	0.38	0.3	0.74
	P-value	0.03	0.04	0.65
*LINC00511*	Expression ratio	0.65	0.56	0.99
	P-value	0.34	0.28	0.99
*MALAT1*	Expression ratio	0.83	0.8	0.92
	P-value	0.7	0.73	0.88
*VDR*	Expression ratio	0.42	0.31	0.93
	P-value	0.05	0.02	0.94

*VDR* expression was significantly associated with sex of patients in a way that most male patients exhibited down-regulation of this gene in their tumor tissue samples compared with the paired ANCTs (P = 0.03). Expressions of *VDR*-associated lncRNAs were not associated with any of clinical or demographic data of patients including age, sex, tumor subtype, stage or history of smoking (Table 3) (see Table 4).

**Table 3:** Association between expression of genes and clinical data (AC: adenocarcinoma, SCC: squamous cell carcinoma).

	*SNHG6* up-regulation	*SNHG6* down-regulation	P value	*SNHG16* up-regulation	*SNHG16* Down-regulation	P value	*LINC00346* up-regulation	*LINC00346* down-regulation	P value	*LINC00511* up-regulation	*LINC00511* down-regulation	P value	*MALAT1* up-regulation	*MALAT1* down-regulation	P value	*VDR* up-regulation	*VDR* down-regulation	P value
Age			0.58			0.78			0.8			0.53			0.36			0.38
>60	7 (31.8%)	15 (68.2%)		9 (40.9%)	13 (59.1%)		8 (36.4%)	14 (63.6%)		12 (54.5%)	10 (45.5%)		13 (59.1%)	9 (40.9%)		6 (27.3%)	16 (72.7%)	
≤60	8 (40%)	12 (60%)		9 (45%)	11 (55%)		8 (40%)	12 (60%)		9 (45%)	11 (55%)		9 (45%)	11 (55%)		8 (40%)	12 (60%)	
Sex			1			0.08			0.68			0.49			0.37			0.03
Female	11 (36.7%)	19 (63.3%)		10 (33.3%)	20 (66.7%)		12 (40%)	18 (60%)		16 (53.3%)	14 (46.7%)		17 (56.7%)	13 (43.3%)		5 (23.3%)	23 (76.7%)	
Male	4 (33.3%)	8 (66.7%)		8 (66.7%)	4 (33.3%)		4 (33.3%)	8 (66.7%)		5 (41.7%)	7 (58.3%)		5 (41.7%)	7 (58.3%)		7 (58.3%)	5 (41.7%)	
Subtype			0.3			0.85			0.58			1			0.37			0.5
AC	7 (29.2%)	17 (70.8%)		10 (41.7%)	14 (58.3%)		10 (41.7%)	14 (58.3%)		12 (50%)	12 (50%)		14 (58.3%)	10 (41.7%)		9 (37.5%)	15 (62.5%)	
SCC	8 (44.4%)	10 (55.6%)		8 (44.4%)	10 (55.6%)		6 (33.3%)	12 (66.7%)		9 (50%)	9 (50%)		8 (44.4%)	10 (55.6%)		5 (27.8%)	13 (72.2%)	
Stage			0.53			0.43			0.47			0.67			0.56			0.11
I	4 (50%)	4 (50%)		5 (62.5%)	3 (37.5%)		4 (50%)	4 (50%)		5 (62.5%)	3 (37.5%)		4 (50%)	4 (50%)		3 (37.5%)	5 (62.5%)	
II	6 (37.5%)	10 (62.5%)		7 (43.8%)	9 (56.2%)		7 (43.8%)	9 (56.3%)		7 (43.8%)	9 (56.3%)		10 (62.5%)	6 (37.5%)		8 (50%)	8 (50%)	
III	5 (27.8%)	13 (72.2%)		6 (33.3%)	12 (66.7%)		5 (27.8%)	13 (72.2%)		9 (50%)	9 (50%)		8 (44.4%)	10 (55.6%)		3 (16.7%)	15 (83.3%)	
Smoking			1			0.25			1			1			0.44			1
Yes	3 (37.5%)	5 (62.5%)		5 (62.5%)	3 (37.5%)		3 (37.5%)	5 (62.5%)		4 (50%)	4 (50%)		3 (37.5%)	5 (62.5%)		3 (37.5%)	5 (62.5%)	
No	12 (35.3%)	22 (64.7%)		13 (38.2%)	21 (61.8%)		13 (38.2%)	21 (61.8%)		17 (50%)	17 (50%)		19 (55.9%)	15 (44.1%)		11 (32.4%)	23 (67.6%)

**Table 4:** Correlation coefficients between expression levels of *VDR* and associated lncRNAs (*denotes P values less than 0.05, ** denotes P values less than 0.01).

Genes		*VDR*	*MALAT1*	*LINC00511*	*LINC00346*	*SNHG16*
*SNHG6*	Tumor Tissues	0.16*	0.39**	0.41**	0.43**	0.69**
	ANCTs	0.001	0.04	0.05	0.05	0.06
*SNHG16*	Tumor Tissues	0.26**	0.39**	0.53**	0.56**	
	ANCTs	0.1*	0.22*	0.31**	0.34**	
*LINC00346*	Tumor Tissues	0.25*	0.5**	0.74		
	ANCTs	0.1*	0.67**	0.83**		
*LINC00511*	Tumor Tissues	0.24*	0.36**			
	ANCTs	0.14*	0.67**			
*MALAT1*	Tumor Tissues	0.2*				
	ANCT	0.02				

### 3.3 ROC curve analysis

**Fig. 2:**
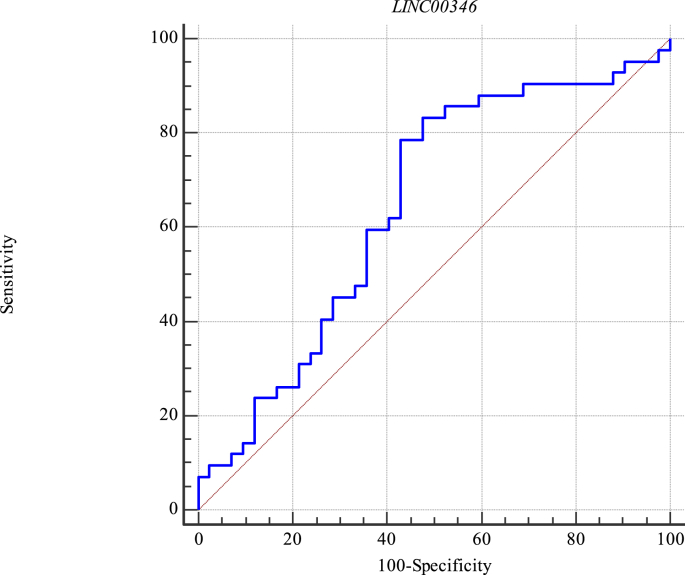
ROC curve for assessment of diagnosis power of *LINC00346* in lung cancer.

Expression levels of *LINC00346* could discriminate lung cancer tissues from ANCTs with sensitivity of 83.3% and specificity of 52.4% (AUC = 0.64, Estimate criterion > - 1. 38, J = 0.35, P value = 0.018). Fig. 2 shows the depicted ROC curve for this lncRNA.

### 3.4 Correlation between expressions of *VDR* and lncRNAs in lung tissues

There were several significant pairwise correlations between expression levels of *VDR* and associated lncRNAs both in tumoral tissues and ANCTs. Notably, correlations between expressions of *SNHG6* and other genes were all significant in tumoral tissues but insignificant in ANCTs (Table 3).

## 4 Discussion

Vitamin D has various biological activities such as anti-proliferative and pro-differentiating roles which have potentiated this vitamin as an anti-cancer agent [10]. In line with these effects, several studies have pointed an inverse correlation between vitamin D levels and risk of lung cancer [11]. Anti-proliferative effects of vitamin D is mediated through its binding with VDR [4]. The observed expression of VDR in the majority of tested lung cancer cells and a significant number of clinical samples has indicated the susceptibility of these cells to the differentiating effects of vitamin D [3]. Notably, expression level of VDR has been associated with differential expression of thousands of genes [12], indicating the presence of an intricate functional network between VDR and other genes. In the present investigation, we appraised expression of *VDR* and five related lncRNAs in lung cancer tissues and ANCTs. LncRNAs have been implicated in the pathogenesis of lung cancer [13]. We detected significant down-regulation of *VDR* in in tumor tissues obtained from male patients compared with their matched ANCTs. However, this pattern was not detected in female subjects. Previous studies reported better survival rate of lung cancer in females compared with males when survival rates have been adjusted for disease stage [14,15]. As nuclear expression of VDR has been linked with better survival in NSCLC [16], the observed different patterns of *VDR* expression between males and females in the current study are in accordance with the better prognosis of lung cancer in females.

Among the assessed lncRNAs was *MALAT1*, a well-recognized oncogenic lncRNA in lung cancer [17]. The association between expression of this lncRNA and *VDR* has been assessed in breast cancer tissues [8]. Here, we detected a modest correlation between expression levels of *MALAT1* and *VDR* in tumor tissues but not in ANCTs. In mouse keratinocytes, *VDR* knockout has resulted in up-regulation of Malalt1 [6,7]. In spite of the acknowledged role of *MALAT1* in the pathogenesis of lung cancer [18], we could not find any significant difference in expression of this lncRNA between lung cancer tissues and ANCTs. The possible explanation for this observation is the influence of tumor microenvironment on the adjacent tissue and the resultant up-regulation of this lncRNA in the histologically normal tissues adjacent to tumor tissues. However, Lin et al. have previously reported up-regulation of this lncRNA in NSCLC tissues compared with ANCTs [19]. The possible difference in the method of surgical excision of ANCTs might be involved in the discrepancy between our results and results of Lin et al. study.

Expression of *LINC00346* was significantly decreased in tumoral tissues compared with ANCTs. When evaluating expression of this lncRNA based on the sex of patients, differences in its expression was only significant among males. A recent study has indicated the oncogenic role of this lncRNA in pancreatic cancer [20]. *LINC0*0346 has sequestered miR-188-3p and obstructed the suppression of BRD4 by miR-188-3p in pancreatic cancer cells [21]. In breast cancer, a significant association has been found between expression of *LINC00346* and tubule formation [8]. The difference in the expression pattern of *LINC00346* in lung and pancreatic cancers might be explained by the presence of tissue-specific targets for this lncRNA.

We detected no significant difference in expression of *SNHG16* between cancerous and non-cancerous tissues. However, Han et al. have shown up-regulation *SNHG16* in line cancer cell lines and clinical samples in correlation with tumor progression and poor prognosis [22]. Such discrepancy might be due to relatively small sample size of our study or the presence of population-specific factors such as environmental exposures that modulate expression of this lncRNA. Future studies are needed to unravel the underlying mechanism of this inconsistent pattern of expression.

Assessment of association between expression of genes and clinical data showed association between *VDR* expression and sex of patients in a way that most male patients exhibited down-regulation of this gene in their tumor tissue samples compared with the paired ANCTs. Correlae et al. have reported sex-based differences in immunomodulatory effects of vitamin D in multiple sclerosis patients and normal controls. However, they reported similar levels of VDR expression between males and females [23]. Future studies are needed to address the mechanism for the observed association between relative expression of *VDR* in tumoral tissues versus ANCTs and sex of lung cancer patients.

Expressions of *VDR*-associated lncRNAs were not associated with any of clinical or demographic data of patients including age, sex, tumor subtype, stage or history of smoking. This lack of association might be explained by the relative small sample size of the study. Thus, further expression assays in larger sample sizes are required to find any possible association between expression level of these lncRNAs and clinical data to find the importance of these lncRNAs in the pathogenesis of lung cancer.

Expression levels of *LINC00346* could discriminate lung cancer tissues from ANCTs with sensitivity of 83.3% and specificity of 52.4%. Therefore, this lncRNA cannot be regarded as a single diagnostic marker for lung cancer. However, it might be incorporated in a putative diagnostic panel to increase the sensitivity of discrimination between cancerous and non-cancerous tissues.

Correlations between expressions of *SNHG6* and other genes were all significant in tumoral tissues but insignificant in ANCTs. Such observation implies construction of a novel interactive network between lncRNAs and *VDR* in the context of lung cancer. Identification of such cancer-specific networks not only helps in recognition of pathogenic events in carcinogenesis but also introduce putative targets for therapeutic interventions.

## 5 Conclusion

Taken together, the current investigation potentiates *VDR* and *LINC00346* as possible participants in the pathogenesis of lung cancer. Future functional studies are required to find the underlying mechanism of their participation in lung cancer. Our study has a limitation regarding lack of assessment of protein levels of VDR in the samples.

## CRediT authorship contribution statement

**Tahereh Gheliji:** Methodology. **Vahid Kholghi Oskooei:** Formal analysis. **Asghar Ashrafi Hafez:** Methodology. **Mohammad Taheri:** Supervision. **Soudeh Ghafouri-Fard:** Supervision, Writing - original draft.

## Declaration of competing interest

The authors declare they have no conflict of interest.
